# Comparative Analysis of Microbial Communities in Fronds and Roots of Three Duckweed Species: *Spirodela polyrhiza*, *Lemna minor*, and *Lemna aequinoctialis*

**DOI:** 10.1264/jsme2.ME20081

**Published:** 2020-07-17

**Authors:** Tomoki Iwashita, Yasuhiro Tanaka, Hideyuki Tamaki, Yasuko Yoneda, Ayaka Makino, Yuka Tateno, Yan Li, Tadashi Toyama, Yoichi Kamagata, Kazuhiro Mori

**Affiliations:** 1 Graduate School of Engineering, University of Yamanashi, 4–3–11 Takeda, Kofu, Yamanashi 400–8511, Japan; 2 Graduate School of Life and Environmental Sciences, University of Yamanashi, 4–4–37 Takeda, Kofu, Yamanashi 400–8510, Japan; 3 Bioproduction Research Institute, AIST, 1–1–1 Higashi, Tsukuba, Ibaraki 305–8566, Japan

**Keywords:** duckweed, microbial community, aquatic plant, *Acidobacteria*, *Armatiomonadetes*, *Verrucomicrobia*

## Abstract

The microbial communities inhabiting the fronds of duckweeds have not been investigated in as much detail as those on the roots. We herein examined the microbial communities in three duckweed species using 16S rRNA amplicon sequencing and compared them to those on the roots. The microbial compositions of the fronds were distinct from those of the roots in the three species. Various types of taxonomic bacteria, including rarely cultivated phyla, *Acidobacteria*, *Armatimonadetes*, and *Verrucomicrobia*, were also isolated from the fronds, but at a slightly lower abundance than those from the roots. These results suggest that duckweed fronds are an alternative source for isolating rare and novel microbes, which may otherwise be recalcitrant to cultivation using conventional strategies.

The subfamily *Lemnoideae*, commonly known as duckweeds, includes five genera: *Landoltia*, *Lemna*, *Spirodela*, *Wolffia*, and *Wolffiella*. It is an aquatic floating plant that is distributed worldwide. The genera *Landoltia*, *Lemna*, and *Spirodela* generally consist of two parts: fronds (fusion of the leaf and stem) and roots, whereas the latter two genera, *Wolffia* and *Wolffiella*, are rootless and composed of fronds only. These plants purify water by absorbing nutrients (nitrogen and phosphorus) and degrading various types of organic matter, including recalcitrant toxic chemical compounds, such as nitrophenols, bisphenols, and nonylphenols (Körner *et al.*, 1998; Toyama *et al.*, 2009; Hoang *et al.*, 2010; Kristanti *et al.*, 2012). Therefore, wastewater treatment systems have been developed using duckweeds (Dalu and Ndamba, 2003; Shi *et al.*, 2010; Priya *et al.*, 2012).

The microbes inhabiting the roots of duckweeds have been investigated because they play a key role in degrading pollutive organic compounds (Yamaga *et al.*, 2010; Kristanti *et al.*, 2012). Recent studies revealed the microbial community diversity and composition of the roots and whole plant body of duckweeds using culture-independent methods (Zhao *et al.*, 2014, 2015; Chen *et al.*, 2019). We also examined the microbial communities associated with the roots of *Spirodela polyrhiza* using both culture-independent and -dependent approaches (Matsuzawa *et al.*, 2010; Tanaka *et al.*, 2018). The findings obtained showed that the roots harbored diverse microbes, including some taxonomically novel bacteria (16S rRNA gene sequence similarity of less than 97% to known species) and rarely cultivated bacterial groups (*e.g.*, *Armatimonadetes* and *Verrucomicrobia*). Additionally, these microbes were readily isolated without extensive efforts, indicating that the roots of duckweeds are sources for the isolation of rare and novel microbes.

Limited information is currently available on the microbes inhabiting the fronds of duckweeds; there has only been one study to date on the fronds of the rootless-type duckweed, *Wolffia australiana* (Xie *et al.*, 2015), which focused on microbial communities analyzed by Illumina HiSeq 2000. Since the fronds of duckweeds float on water and interact with microbes in water, unique microbes may be associated with the fronds. Therefore, they may contribute to the purification of water in the environment. In the present study, we investigated microbes on the fronds of three duckweed species, *S. polyrhiza*, *Lemna minor*, and *Lemna aequinoctialis*, which are often used in water purification studies (Toyama *et al.*, 2009; Hoang *et al.*, 2010; Li *et al.*, 2014), using bacterial 16S rRNA gene amplicon sequencing, and compared the data obtained with those on the roots. Additionally, microbial isolation from frond samples was performed to verify the usefulness of the fronds of duckweeds as a better source of novel or rarely cultivated microbes than the roots.

Three species of duckweeds (*S. polyrhiza*, *L. minor*, and *L. aequinoctialis*) grown in a pond located within the Yamanashi prefectural wood park “Kanegawa-no-mori” (Fuefuki, Yamanashi, Japan; 35°38′23″ N, 138°40′36″ E) and a pond water sample near the plants were collected in August 2013. Duckweed samples (*S. polyrhiza*; three plants, *L. minor*, and *L. aequinoctialis*; 10 plants) were gently washed twice with 30‍ ‍mL of sterilized DTS medium (Matsuzawa *et al.*, 2010) in a 50-mL conical tube. After washing, each duckweed was divided into the frond and root parts by cutting them off with a sterilized scalpel. These parts were subjected to total DNA extraction using Cica Geneus DNA Extraction Reagent (Kanto Chemical). The pond water sample (100‍ ‍mL) was filtrated using a membrane filter with a pore size of 0.22‍ ‍μm (Omnipore; Merck), and the microbes trapped on the filter were suspended in 500‍ ‍μL of TE buffer. DNA extraction from a portion (100‍ ‍μL) of this suspension was also conducted using Cica Geneus DNA Extraction Reagent. The extracted DNAs from all samples were purified using Zymo-Spin (Zymo Research) and then subjected to PCR using Eub-515F (5′-ACACTCTTTCCCTACACGACGCTCTTCCGATCTGTGCCAGCMGCCGCGGTAA-3′; the sequence for 2nd PCR is underlined), and Eub-806R (5′-GTGACTGGAGTTCAGACGTGTGCTCTTCCGATCTGGACTACHVGGGTWTCTAAT-3′; the sequence for 2nd PCR is underlined) for the amplification of the 16S rRNA gene fragment (V4 region) as previously described (Shrestha *et al.*, 2017). The preparation and sequencing of 2nd PCR amplicons using the MiSeq sequencer (Illumina) were completed by FASMAC (Atsugi). The operational taxonomic units (OTUs) obtained, based on a threshold of 97% similarities, were classified into either the phylum or family level. Sequences were deposited in the DNA data bank of Japan under the accession number DRA009780. All statistical analyses were conducted using R (version 3.5.0). A heat map was created using the gplots package (3.0.1), and a cluster analysis was also performed using the dist function “Euclidean” and the average method. A principal component analysis (PCA) was conducted using the function “prcomp.”

A low-nutrient medium, DTS (pH 7.0) medium solidified with 1.5% agar, was used for microbial isolation. Duckweed plants (three *S. polyrhiza* plants, five *L. minor* plants, and five *L. aequinoctialis* plants) were washed twice with 30‍ ‍mL of sterilized DTS medium. After washing, the fronds and roots were separated by cutting them with a sterilized scalpel. They were then homogenized with 10‍ ‍mL of sterilized DTS medium using the Vibra-Cell Ultrasonic Liquid Processor VCX 130 (130 W, 20 kHz) (Sonics) for 1‍ ‍minute (roots) or 2‍ ‍minutes (fronds). The homogenates and pond water sample were diluted 10^–1^ to 10^–4^-fold with DTS medium. Each diluted sample (50‍ ‍μL) was independently inoculated on DTS agar (1.5%) plates in triplicate and incubated at 25°C for 30 days. The 16S rRNA genes of isolates were amplified by a colony direct PCR method using Eub-8F (5′-AGAGTTTGATCMTGGCTCAG-3′) and Eub-1512R (5′-ACGGYTACCTTGTTACGACTT-3′) primers (Weisburg *et al.*, 1991; Kane *et al.*, 1993). Amplified DNAs were subjected to a RFLP analysis using two types of restriction endonucleases *Hha*I and *Hae*III (Takara). The 16S rRNA gene fragments from representative isolates of each RFLP group were purified using the Cica Geneus PCR & Gel Prep Kit (Kanto Chemical) and sequenced as previously described (Tamaki *et al.*, 2005). Sequence data (the GenBank/EMBL/DDBJ accession numbers LC523912–LC523985) were compared with those present in the EzBioCloud database (https://www.ezbiocloud.net/). Diversity in bacterial abundance at the level of OTUs was evaluated using the calculation for Hurlbert’s PIE (probability of an interspecific encounter) index [(*PIE*)={*N*/(*N*–1)}{1–Σ(*pi*)^2^}], where *N* is the total number of OTUs and *pi* is the proportion of OTUs (Hurlbert, 1971).

The sequencing of 16S rRNA gene amplicons from the fronds and roots of three species of duckweeds and the pond water sample taken from near the plant samples yielded a total of 671,877 sequences. These sequences were subsequently classified into 7,744 bacterial OTUs. The numbers of total OTUs and specific OTUs in each sample are shown in Table S1. At the phylum level, OTUs were classified into 53 different taxonomic groups, 11 of which were distributed in at least one plant or water sample by more than 1.0% (Fig. 1A). Among the 11 phyla, the phylum *Proteobacteria* was the most predominant group in all samples (fronds: 57.3%–62.4%, roots: 48.1%–59.6%, and pond water: 43.3%). However, the other constituents between plant samples and the pond water sample differed; seven and nine phyla, except for *Proteobacteria*, were detected in the root and frond samples, respectively, while only four phyla were found in the pond water.

Since differences in microbial communities between the frond and root samples at the phylum level were unclear, we examined communities at the family level. In total, 478 bacterial families were observed, and 108 of the families were distributed above 0.1% in at least one sample, as shown in Fig. 1B. Within these families, 68–72 groups (72 *S. polyrhiza*, 68 *L. minor*, and 70 *L. aequinoctialis*) and 67–76 groups (76 *S. polyrhiza*, 67 *L. minor*, and 75 *L. aequinoctialis*) were found in frond and root samples, respectively. In contrast, in the water sample, only 35 groups showed abundance >0.1%. Based on the proportions of the prominent families (108 families) in each sample, the resemblance of the microbial community was evaluated using a hierarchical cluster heat map analysis and PCA analy­sis. The bacterial communities of plant samples markedly differed from those of the water sample (Fig. S1 and S2). The results obtained also revealed that frond and root samples were clustered into two separate groups, suggesting that the bacterial communities on the fronds were distinct from those on the roots, independent of species differences between duckweeds. Within the families shown in Fig. S1, 13 families on the fronds and 11 families on the roots showed abundance >1.0% in each sample. Of these, *Moraxellaceae* and Unclassified Solibacterales 2 were frequently detected only in the fronds and roots, respectively, suggesting that these microbial groups are candidate core microbes for each plant part. Although the reason for differences in microbial communities between frond and root samples currently remains unclear, it may be due to chemical and physical complex factors, such as differences in the compositions of exudates, surface structures, and surrounding factors that affect the metabolism of the plants (*e.g.*, CO_2_, O_2_, light radiation, and water availability).

Based on bacterial abundance at the level of OTUs, bacterial diversity in each sample was evaluated using the PIE index, which is unbiased by sampling size. No marked differences were observed in diversity between frond and root samples in all duckweeds; however, the PIE index was higher than that in pond water (Table S1). In terrestrial plants, the richness and diversity of bacterial communities inhabiting the phyllosphere are lower than those in roots or the rhizosphere (Bodenhausen *et al.*, 2013; Wagner *et al.*, 2016). However, the present results showed that this may not be the case for the fronds and roots in duckweeds. This may simply be because both the fronds and roots of duckweeds are on or in water; in terrestrial plants, the phyllosphere is in air, whereas the rhizosphere is in soil, which harbors a greater diversity of microbes than air. Therefore, a wide variety of microbes in water have a chance to interact evenly with and attach to the two plant parts.

To confirm whether the fronds of duckweeds are also a useful isolation source of novel microbes in addition to the roots (Matsuzawa *et al.*, 2010; Tanaka *et al.*, 2018), we cultivated microbes associated with the fronds and roots of duckweeds. Twenty to thirty colonies were randomly selected from DTS agar plates, which were independently inoculated with homogenates of the plant samples or pond water. The 16S rRNA genes of these colonies were amplified by PCR and grouped into phylotypes by a RFLP analysis. The isolates from duckweeds were grouped into 13–25 phylotypes for frond samples (30 strains each from *S. polyrhiza*, *L. minor*, and *L. aequinoctialis* were divided into 25, 14, and 13 phylotypes, respectively) and 15–20 phylotypes for root samples (30, 27, and 21 strains from *S. polyrhiza*, *L. minor*, and *L. aequinoctialis* were divided into 20, 20, and 15 phylotypes, respectively). In contrast, 30 isolates from pond water were composed of 11 phylotypes. The 16S rRNA gene sequences of the representative phylotypes were compared with those in the EzBioCloud database (Table 1); phylogenetic distribution at the phylum level is shown in Fig. S3. All isolates were classified into seven phyla, and the most predominant phylum was *Proteobacteria* in all samples, similar to the results of the culture-independent analysis. Members of the rarely cultivated bacterial groups, *Acidobacteria*, *Armatimonadetes*, and *Verrucomicrobia* were isolated in the present study, but only from the duckweed samples (not from pond water). Three bacterial strains were from frond samples (Acidobacteria bacterium strain 5-B1; *S. polyrhiza*, Armatimonadetes bacterium strain C6, and Verrucomicrobia bacterium strain 5-B3; *L. aequinoctialis*), while two strains were from root samples (Acidobacteria bacterium strain 5-A6; *L. minor* and Verrucomicrobia bacterium strain 4-F7; *S. polyrhiza*). Among these rarely cultivated microbes, the most interesting isolate was Armatimonadetes bacterium strain C6, from the *L. aequinoctialis* frond, because only seven strains in this phylum have been isolated to date: the roots of aquatic plants; three strains, geothermally heated soil; two strains, ginseng field soil; one strain, and the trunk surface of a tree; one strain (Lee *et al.*, 2011; Tamaki *et al.*, 2011; Im *et al.*, 2012; Tanaka *et al.*, 2018; Li *et al.*, 2019). We previously isolated three strains of this phylum from aquatic plant root samples. One strain was from the root of wild reed, and the others were from the root of laboratory-grown *S. polyrhiza*, which was inoculated with homogenates of Japanese loosestrife root (Tamaki *et al.*, 2011; Tanaka *et al.*, 2018). With the inclusion of strain C6 isolated in the present study, 50% of the *Armatimonadetes* isolates (four out of eight strains) obtained to date have been derived from aquatic plant-related samples, suggesting that microbes within this distinct phylum have a specific niche in which to thrive. Therefore, it may be possible to streamline the isolation of this elusive taxon using aquatic environment samples, thereby gaining further insights into the ecophysiological properties of microbes within this particular phylum.

The taxonomic novelty of all isolates was evaluated using the criterion that an isolate with less than 97% 16S rRNA gene sequence similarity to any known bacterial species was defined as a phylogenetically novel bacterium. Among the isolates derived from the root samples, 57, 44, and 67% of isolates from *S. polyrhiza*, *L. minor*, and *L. aequinoctialis* were taxonomically novel. The proportion of novel bacterial isolates from the root of *S. polyrhiza* was consistent with previous findings (Matsuzawa *et al.*, 2010). The scores of the root for *L. minor* and *L. aequinoctialis* were similar to that for *S. polyrhiza*, demonstrating that novel bacteria may also be obtained from the roots of *L. minor* and *L. aequinoctialis* in addition to *S. polyrhiza*. In contrast, the proportions of novel bacterial isolates from frond samples were slightly lower than those from root isolates; the scores were 30% in *S. polyrhiza*, 23% in *L. minor*, and 37% in *L. aequinoctialis*, even though, these scores were markedly higher than the isolates from pond water (7%). Five RFLP groups (Nos. 10, 42, 71, 72, and 74) composed of taxonomically novel bacterial isolates, including the rarely cultivated bacterial phyla, *Armatimonadetes*, *Acidobacteria*, and *Verrucomicrobia*, were specifically obtained from frond samples (Table 1). These results indicate that the fronds of duckweeds are useful sources for isolating a wide variety of novel microbes as well as their roots.

We previously reported a new microbial isolation method using the interaction between duckweed and microbes, which is referred to as the “duckweed-microbe co-cultivation method” (Tanaka *et al.*, 2018). Using this method, we inoculated microcosms from an environmental sample into aseptic duckweeds. We co-cultivated them for two weeks, allowing a variety of novel microbes to grow on the surface of the root. Therefore, we concluded that using this method, the entire duckweed body (the frond as well as the root) may be a suitable substratum to enrich and isolate yet-to-be cultured, but ecologically and practically important microorganisms.

## Citation

Iwashita, T., Tanaka, Y., Tamaki, H., Yoneda, Y., Makino, A., Tateno, Y., et al. (2020) Comparative Analysis of Microbial Communities in Fronds and Roots of Three Duckweed Species: *Spirodela polyrhiza*, *Lemna minor*, and *Lemna aequinoctialis*. *Microbes Environ ***35**: ME20081.

https://doi.org/10.1264/jsme2.ME20081

## Supplementary Material

Supplementary Material

## Figures and Tables

**Fig. 1.**
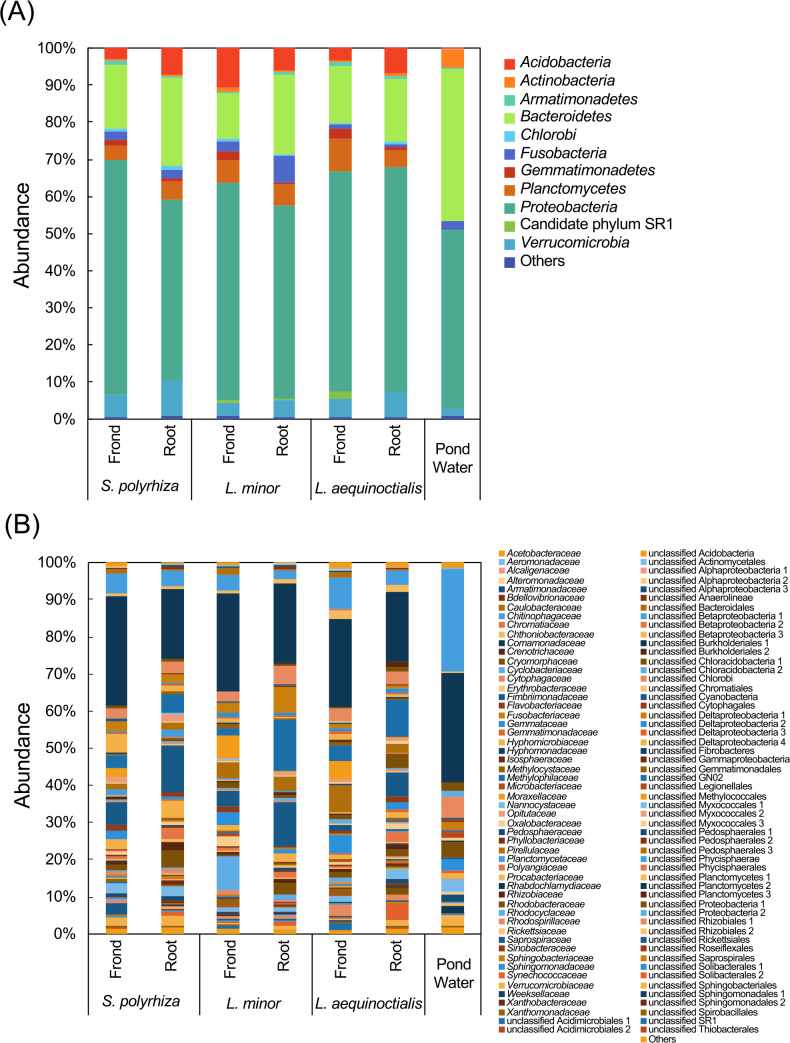
Microbial compositions in duckweed fronds, roots, and pond water at the level of the phylum (A) and family (B). Sequences of taxa with maximum abundance <1.0% for phylum (A) and <0.1% for family (B) in each sample were assembled as “Others”.

**Table 1.** Phylogenetic classification of isolates based on 16S rRNA gene sequences

RFLP Group	No. of isolates							Closest species (Accession number)	Phylum (Class)	Similarity (%)	Compared length (bp)
	*S. polyrhiza*		*L. minor*		*L. aequinoctialis*		Pond water				
	fronds	roots	fronds	roots	fronds	roots					
**1**	**2**	**4**	**6**	**3**	**8**	**3**		***Oharaeibacter diazotrophicus***** (LC153750)**	***Proteobacteria***** (Alpha)**	**96**	**701**
2	2	1						*Polymorphobacter fuscus* (KF737330)	*Proteobacteria* (Alpha)	100	694
**3**	**2**	**1**	**1**	**1**		**1**		***Phreatobacter oligotrophus***** (HE616165)**	***Proteobacteria***** (Alpha)**	**94**	**775**
4	2	1						*Hyphomicrobium aestuarii* (Y14304)	*Proteobacteria* (Alpha)	98	773
5	1		1	1				*Phenylobacterium conjunctum* (AJ227767)	*Proteobacteria* (Alpha)	100	751
6	1							*Novosphingobium aquiterrae* (FJ772064)	*Proteobacteria* (Alpha)	99	756
7	1		1	1				*Novosphingobium piscinae* (LK056647)	*Proteobacteria* (Alpha)	100	741
8	1							*Carbophilus carboxidus* (JN175336)	*Proteobacteria* (Alpha)	99	759
9	1						1	*Aquidulcibacter paucihalophilus* (NCSQ01000081)	*Proteobacteria* (Alpha)	100	761
**10**	**1**							***Methylocapsa aurea***** (JQKO01000009)**	***Proteobacteria***** (Alpha)**	**96**	**780**
**11**		**2**		**1**				***Rhodobacter sediminis***** (LT009496)**	***Proteobacteria***** (Alpha)**	**96**	**763**
**12**		**1**						***Phreatobacter oligotrophus***** (HE616165)**	***Proteobacteria***** (Alpha)**	**93**	**767**
13			2		1	1		*Sphingomonas pituitosa* (AJ243751)	*Proteobacteria* (Alpha)	99	770
14			2	1	1			*Rhizobium esperanzae* (KC293513)	*Proteobacteria* (Alpha)	99	688
15			1	1	1			*Caulobacter segnis* (CP002008)	*Proteobacteria* (Alpha)	100	739
16			1					*Caulobacter segnis* (CP002008)	*Proteobacteria* (Alpha)	100	777
17			1					*Devosia enhydra* (jgi.1047208)	*Proteobacteria* (Alpha)	97	771
**18**				**3**		**1**		***Hyphomicrobium nitrativorans***** (CP006912)**	***Proteobacteria***** (Alpha)**	**92**	**777**
19				1				*Mesorhizobium chacoense* (AJ278249)	*Proteobacteria* (Alpha)	99	769
20				1				*Devosia confluentis* (KU507536)	*Proteobacteria* (Alpha)	98	751
21				1			5	*Sediminicoccus rosea* (JX294477)	*Proteobacteria* (Alpha)	100	731
22				1				*Novosphingobium lentum* (BCTW01000008)	*Proteobacteria* (Alpha)	99	745
23				1				*Phreatobacter oligotrophus* (HE616165)	*Proteobacteria* (Alpha)	100	775
**24**				**1**				***Phreatobacter oligotrophus***** (HE616165)**	***Proteobacteria***** (Alpha)**	**94**	**780**
**25**					**1**	**2**		***Methylovirgula ligni***** (FM252034)**	***Proteobacteria***** (Alpha)**	**94**	**776**
26					1			*Mesorhizobium chacoense* (AJ278249)	*Proteobacteria* (Alpha)	99	780
27						2		*Ensifer morelensis* (AY024335)	*Proteobacteria* (Alpha)	98	773
**28**						**1**		***Sphingomonas silvisoli***** (KU597283)**	***Proteobacteria***** (Alpha)**	**96**	**780**
**29**						**1**		***Oharaeibacter diazotrophicus***** (LC153750)**	***Proteobacteria***** (Alpha)**	**89**	**792**
30		1					1	*Oharaeibacter diazotrophicus* (LC153750)	*Proteobacteria* (Alpha)	97	765
31							3	*Novosphingobium fuchskuhlense* (KQ954244)	*Proteobacteria* (Alpha)	100	740
32							2	*Gemmobacter straminiformis* (KX832992)	*Proteobacteria* (Alpha)	99	693
33	2							*Ideonella dechloratans* (X72724)	*Proteobacteria* (Beta)	97	807
34	1	3	1	1				*Aquabacterium olei* (KC424519)	*Proteobacteria* (Beta)	98	551
35	1	4	9	3	2	2		*Rubrivivax gelatinosus* (D16213)	*Proteobacteria* (Beta)	98	730
36	1	1		2				*Pelomonas puraquae* (AM501439)	*Proteobacteria* (Beta)	100	741
37	1	1						*Leptothrix cholodnii* (X97070)	*Proteobacteria* (Beta)	97	745
**38**	**1**	**1**						***Herbaspirillum seropedicae***** (CP011930)**	***Proteobacteria***** (Beta)**	**90**	**769**
39	1							*Aquabacterium commune* (AF035054)	*Proteobacteria* (Beta)	98	780
40	1				7			*Sphaerotilus montanus* (EU636006)	*Proteobacteria* (Beta)	100	773
41	1						10	*Piscinibacterium candidicorallinum* (LT158233)	*Proteobacteria* (Beta)	100	745
**42**	**1**							***Accumulibacter phosphatis***** (CP001715)**	***Proteobacteria***** (Beta)**	**91**	**752**
**43**		**1**						***Thiobacter subterraneus***** (AB180657)**	***Proteobacteria***** (Beta)**	**91**	**808**
44		1						*Curvibacter delicatus* (BCWP01000019)	*Proteobacteria* (Beta)	97	818
45			2					*Ramlibacter henchirensis* (AF439400)	*Proteobacteria* (Beta)	97	787
46			1					*Hydrogenophaga defluvii* (AJ585993)	*Proteobacteria* (Beta)	99	741
47			1					*Piscinibacter aquaticus* (DQ664244)	*Proteobacteria* (Beta)	99	811
**48**				**1**				***Azoarcus buckelii***** (AJ315676)**	***Proteobacteria***** (Beta)**	**92**	**787**
49					2	1		*Methylophilus quaylei* (AY772089)	*Proteobacteria* (Beta)	100	790
**50**						**1**		***Methylotenera versatilis***** (CP002056)**	***Proteobacteria***** (Beta)**	**95**	**821**
**51**						**1**		***Methylotenera mobilis***** (CP001672)**	***Proteobacteria***** (Beta)**	**96**	**811**
52							2	*Curvibacter delicatus* (BCWP01000019)	*Proteobacteria* (Beta)	97	779
53	1							*Silanimonas lenta* (AUBD01000017)	*Proteobacteria* (Gamma)	97	685
54	1							*Tahibacter aquaticus* (AM981201)	*Proteobacteria* (Gamma)	99	687
**55**		**1**						***Lamprocystis roseopersicina***** (AJ006063)**	***Proteobacteria***** (Gamma)**	**90**	**835**
**56**		**2**						***Thioprofundum lithotrophicum***** (AB468957)**	***Proteobacteria***** (Gamma)**	**92**	**807**
57							1	*Rheinheimera aquatica* (GQ168584)	*Proteobacteria* (Gamma)	99	657
**58**	**1**	**1**						***Nemorincola caseinilytica***** (KY233199)**	***Bacteroidetes***	**94**	**782**
59	1							*Parasediminibacterium paludis* (HQ231219)	*Bacteroidetes*	99	730
**60**		**1**		**1**				***Runella palustris***** (KT273904)**	***Bacteroidetes***	**96**	**783**
**61**		**1**						***Solitalea koreensis***** (EU787448)**	***Bacteroidetes***	**82**	**810**
62					1			*Sediminibacterium aquarii* (KR812546)	*Bacteroidetes*	98	759
**63**						**2**		***Rudanella lutea***** (ARPG01000002)**	***Bacteroidetes***	**91**	**779**
**64**						**1**		***Flavitalea gansuensis***** (GU295962)**	***Bacteroidetes***	**95**	**761**
65							3	*Flavobacterium cheonhonense* (GU295972)	*Bacteroidetes*	99	731
**66**							**1**	***Flavobacterium terrae***** (jgi.1107701)**	***Bacteroidetes***	**95**	**791**
**67**							**1**	***Nemorincola caseinilytica***** (KY233199)**	***Bacteroidetes***	**92**	**768**
68						1		*Microbacterium lacus* (AB286030)	*Actinobacteria*	100	690
69					3			*Staphylococcus epidermidis* (L37605)	*Firmicutes*	100	765
**70**		**1**						***Opitutus terrae***** (CP001032)**	***Verrucomicrobia***	**95**	**799**
**71**					**1**			***Prosthecobacter dejongeii***** (U60012)**	***Verrucomicrobia***	**82**	**809**
**72**	**1**							***Aridibacter nitratireducens***** (KX443571)**	***Acidobacteria***	**96**	**826**
**73**				**1**				***Bryobacter aggregatus***** (JNIF01000003)**	***Acidobacteria***	**89**	**740**
**74**					**1**			***Fimbriimonas ginsengisoli***** (CP002763)**	***Armatimonadetes***	**91**	**765**
Total	30	30	30	27	30	21	30				
Novel bacteria	9	17	7	12	11	14	2				

Taxonomically novel bacteria are shown in bold.
