# Supplementary material for: Comparative Analysis of Microbial Communities in Fronds and Roots of Three Duckweed Species: Spirodela polyrhiza, Lemna minor, and Lemna aequinoctialis
Source: Microbes Environ. 2020 Jul 17;35(3):ME20081. doi: 10.1264/jsme2.ME20081 (PMC7511783; doi:10.1264/jsme2.ME20081)
Supplement: Supplementary file 1 — Supplementary Material [file 35_20081_s1.pdf]

Table S1. Total number of sequences, OTU and diversity index on the basis of 16S rRNA amplicon analysis

Sample	*S. polyrhiza*		*L. minor*		*L. aequinoctialis*		Pond water
	fronds	roots	fronds	roots	fronds	roots	
Total sequences	79,776	117,708	105,574	99,732	105,003	84,173	79,911
OTU	1,971	2,680	1,580	2,351	1,537	1,341	1,986
Specific OTU	602	1,095	648	848	714	587	1,146
PIE index	0.962	0.981	0.973	0.978	0.956	0.961	0.915

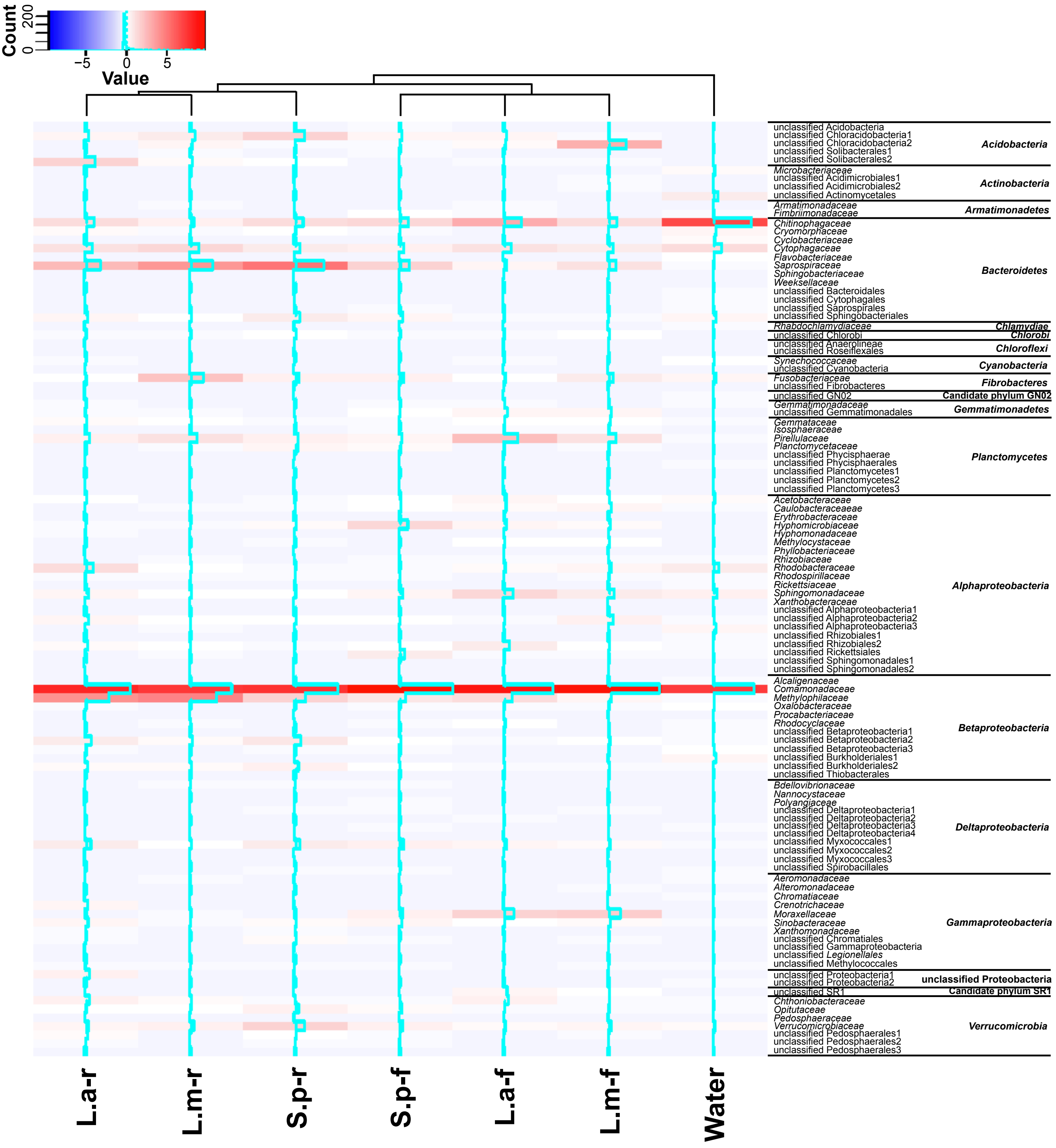

Fig. S1
Iwashita et al.

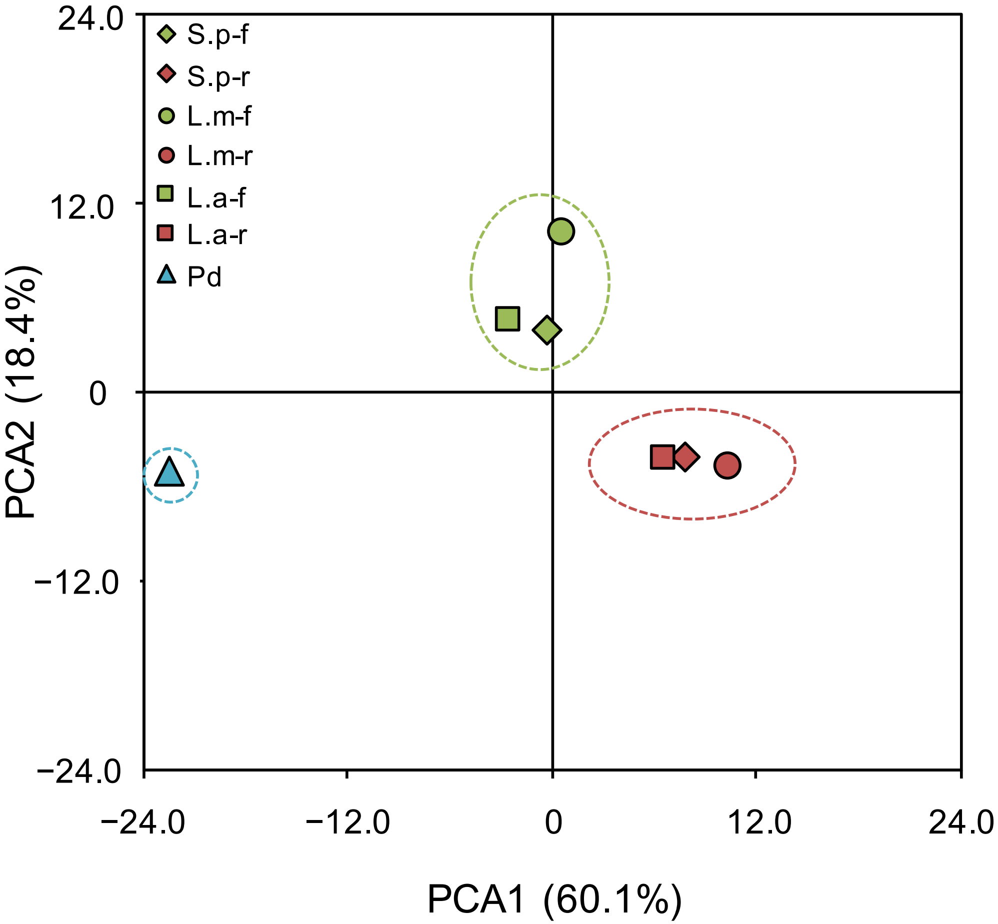

Fig. S2
Iwashita et al.

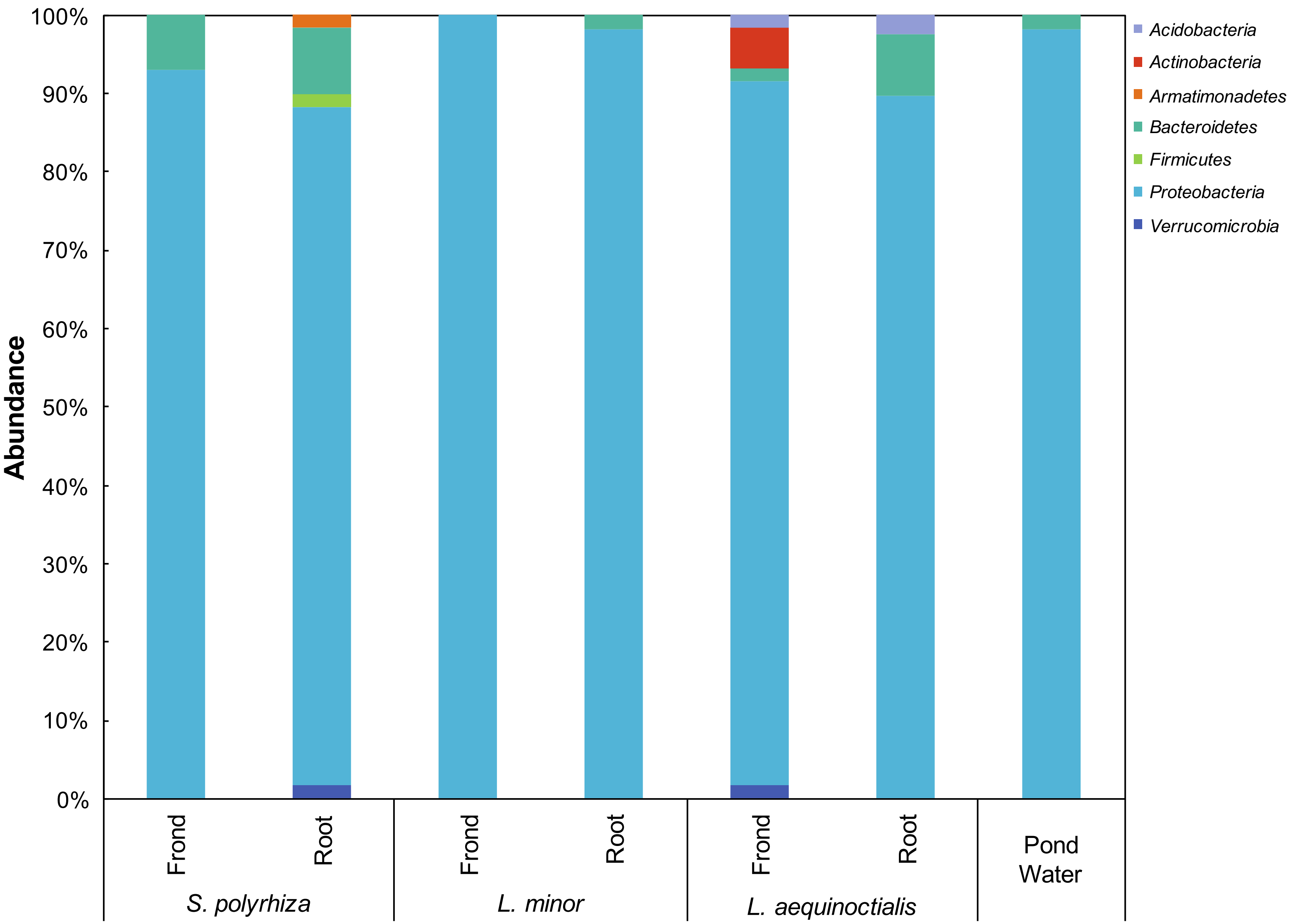

Fig. S3
Iwashita et al.

Fig. S1. Heat map for distribution of bacterial families in duckweed fronds, roots and pond water. Each sample name indicates as follows: S.p-f, fronds of *S. polyrhiza*; S.p-r, roots of *S. polyrhiza*; L.m-f, fronds of *L. minor*; L.m-r, roots of *L. minor*; L.a-f, fronds of *L. aequinoctialis*, L.a-r, roots of *L. aequinoctialis*; Water, pond water.

Fig. S2. Principal component analysis (PCA) based on microbial communities at the family level in duckweed fronds, roots, and pond water (abundance >0.1% in any samples).

Fig. S3. Phylogenetic distribution of the isolates from duckweed fronds, roots and the pond water at the phylum level.
